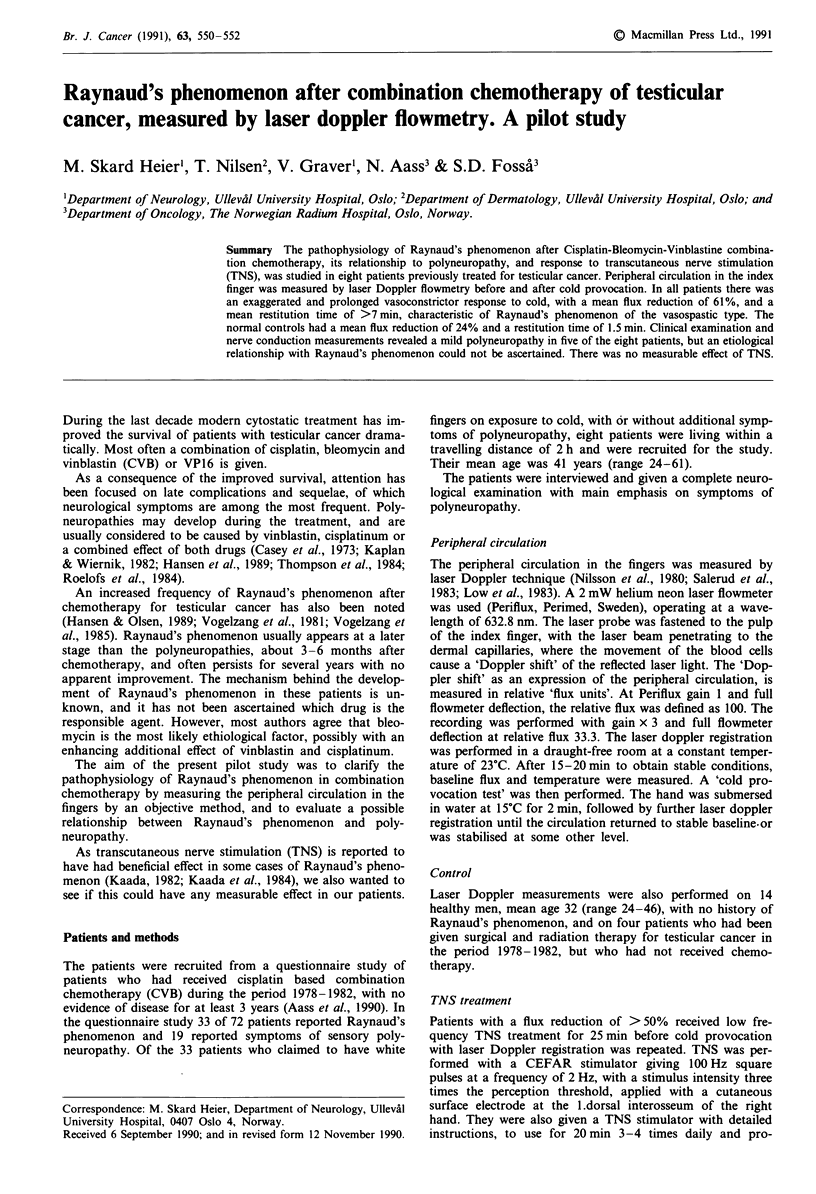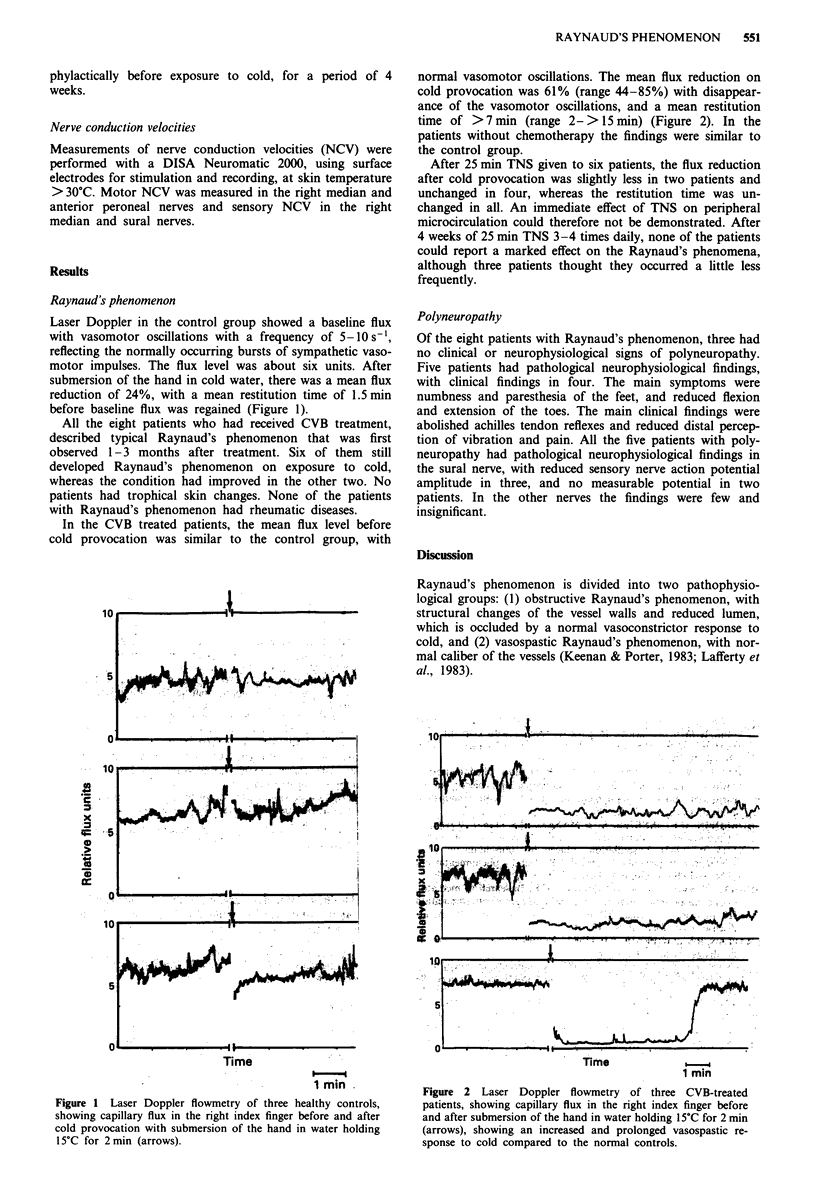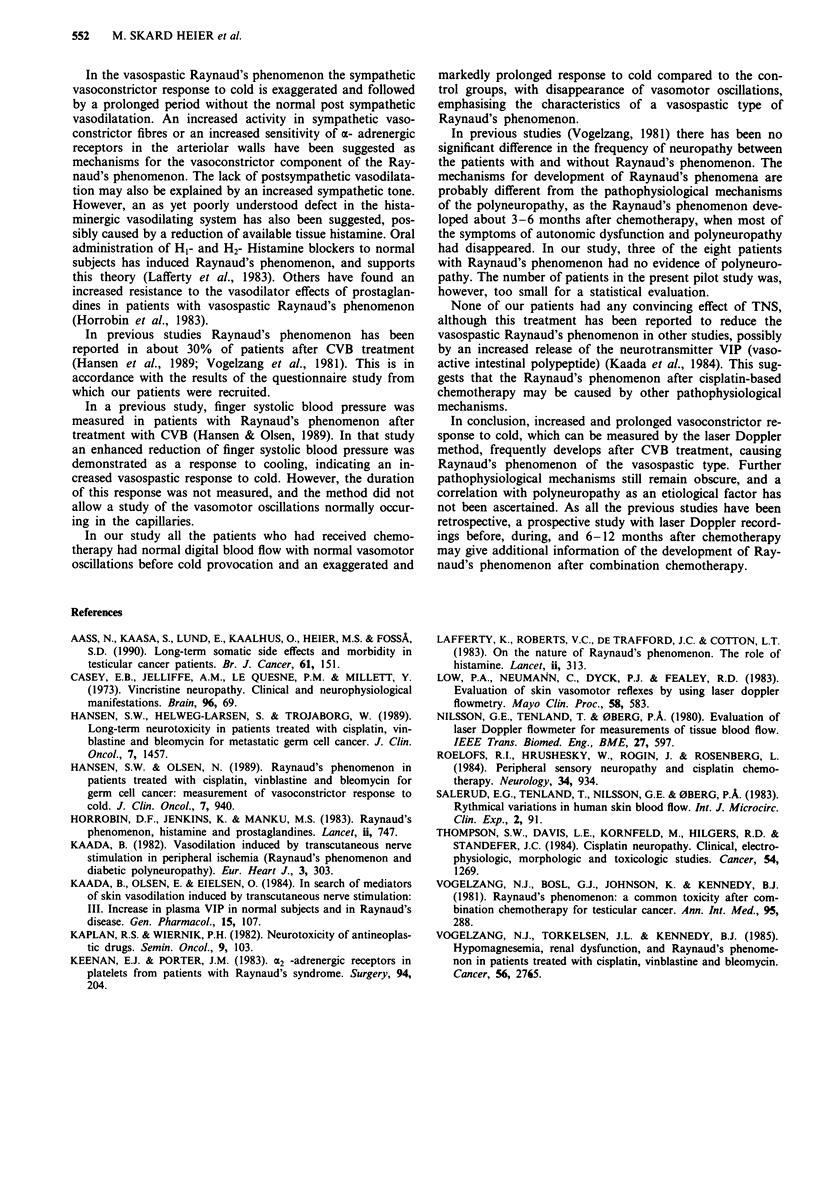# Raynaud's phenomenon after combination chemotherapy of testicular cancer, measured by laser Doppler flowmetry. A pilot study.

**DOI:** 10.1038/bjc.1991.129

**Published:** 1991-04

**Authors:** M. S. Heier, T. Nilsen, V. Graver, N. Aass, S. D. Fosså

**Affiliations:** Department of Neurology, Ullevål University Hospital, Oslo, Norway.

## Abstract

The pathophysiology of Raynaud's phenomenon after Cisplatin-Bleomycin-Vinblastine combination chemotherapy, its relationship to polyneuropathy, and response to transcutaneous nerve stimulation (TNS), was studied in eight patients previously treated for testicular cancer. Peripheral circulation in the index finger was measured by laser Doppler flowmetry before and after cold provocation. In all patients there was an exaggerated and prolonged vasoconstrictor response to cold, with a mean flux reduction of 61%, and a mean restitution time of greater than 7 min, characteristic of Raynaud's phenomenon of the vasospastic type. The normal controls had a mean flux reduction of 24% and a restitution time of 1.5 min. Clinical examination and nerve conduction measurements revealed a mild polyneuropathy in five of the eight patients, but an etiological relationship with Raynaud's phenomenon could not be ascertained. There was no measurable effect of TNS.


Br. J. Cancer (1991), 63, 550-552 © Macmillan Press Ltd., 1991

### Raynaud's phenomenon after combination chemotherapy of testicular cancer, measured by laser doppler flowmetry. A pilot study

M. Skard Heier[1], T. Nilsen[2], V. Graver[1], N. Aass[3] & S.D. Fosså[3]

[1]*Department of Neurology, Ullevål University Hospital, Oslo;* [2]*Department of Dermatology, Ullevål University Hospital, Oslo; and* [3]*Department of Oncology, The Norwegian Radium Hospital, Oslo, Norway.*

**Summary** The pathophysiology of Raynaud's phenomenon after Cisplatin-Bleomycin-Vinblastine combination chemotherapy, its relationship to polyneuropathy, and response to transcutaneous nerve stimulation (TNS), was studied in eight patients previously treated for testicular cancer. Peripheral circulation in the index finger was measured by laser Doppler flowmetry before and after cold provocation. In all patients there was an exaggerated and prolonged vasoconstrictor response to cold, with a mean flux reduction of 61%, and a mean restitution time of >7 min, characteristic of Raynaud's phenomenon of the vasospastic type. The normal controls had a mean flux reduction of 24% and a restitution time of 1.5 min. Clinical examination and nerve conduction measurements revealed a mild polyneuropathy in five of the eight patients, but an etiological relationship with Raynaud's phenomenon could not be ascertained. There was no measurable effect of TNS.

During the last decade modern cytostatic treatment has improved the survival of patients with testicular cancer dramatically. Most often a combination of cisplatin, bleomycin and vinblastin (CVB) or VP16 is given.

As a consequence of the improved survival, attention has been focused on late complications and sequelae, of which neurological symptoms are among the most frequent. Polyneuropathies may develop during the treatment, and are usually considered to be caused by vinblastin, cisplatinum or a combined effect of both drugs (Casey *et al.*, 1973; Kaplan & Wiernik, 1982; Hansen *et al.*, 1989; Thompson *et al.*, 1984; Roelofs *et al.*, 1984).

An increased frequency of Raynaud's phenomenon after chemotherapy for testicular cancer has also been noted (Hansen & Olsen, 1989; Vogelzang *et al.*, 1981; Vogelzang *et al.*, 1985). Raynaud's phenomenon usually appears at a later stage than the polyneuropathies, about 3-6 months after chemotherapy, and often persists for several years with no apparent improvement. The mechanism behind the development of Raynaud's phenomenon in these patients is unknown, and it has not been ascertained which drug is the responsible agent. However, most authors agree that bleomycin is the most likely ethiological factor, possibly with an enhancing additional effect of vinblastin and cisplatinum.

The aim of the present pilot study was to clarify the pathophysiology of Raynaud's phenomenon in combination chemotherapy by measuring the peripheral circulation in the fingers by an objective method, and to evaluate a possible relationship between Raynaud's phenomenon and polyneuropathy.

As transcutaneous nerve stimulation (TNS) is reported to have had beneficial effect in some cases of Raynaud's phenomenon (Kaada, 1982; Kaada *et al.*, 1984), we also wanted to see if this could have any measurable effect in our patients.

### Patients and methods

The patients were recruited from a questionnaire study of patients who had received cisplatin based combination chemotherapy (CVB) during the period 1978-1982, with no evidence of disease for at least 3 years (Aass *et al.*, 1990). In the questionnaire study 33 of 72 patients reported Raynaud's phenomenon and 19 reported symptoms of sensory polyneuropathy. Of the 33 patients who claimed to have white fingers on exposure to cold, with or without additional symptoms of polyneuropathy, eight patients were living within a travelling distance of 2 h and were recruited for the study. Their mean age was 41 years (range 24-61).

The patients were interviewed and given a complete neurological examination with main emphasis on symptoms of polyneuropathy.

### *Peripheral circulation*

The peripheral circulation in the fingers was measured by laser Doppler technique (Nilsson *et al.*, 1980; Salerud *et al.*, 1983; Low *et al.*, 1983). A 2 mW helium neon laser flowmeter was used (Periflux, Perimed, Sweden), operating at a wavelength of 632.8 nm. The laser probe was fastened to the pulp of the index finger, with the laser beam penetrating to the dermal capillaries, where the movement of the blood cells cause a 'Doppler shift' of the reflected laser light. The 'Doppler shift' as an expression of the peripheral circulation, is measured in relative 'flux units'. At Periflux gain 1 and full flowmeter deflection, the relative flux was defined as 100. The recording was performed with gain × 3 and full flowmeter deflection at relative flux 33.3. The laser doppler registration was performed in a draught-free room at a constant temperature of 23°C. After 15-20 min to obtain stable conditions, baseline flux and temperature were measured. A 'cold provocation test' was then performed. The hand was submersed in water at 15°C for 2 min, followed by further laser doppler registration until the circulation returned to stable baseline or was stabilised at some other level.

### *Control*

Laser Doppler measurements were also performed on 14 healthy men, mean age 32 (range 24-46), with no history of Raynaud's phenomenon, and on four patients who had been given surgical and radiation therapy for testicular cancer in the period 1978-1982, but who had not received chemotherapy.

### *TNS treatment*

Patients with a flux reduction of >50% received low frequency TNS treatment for 25 min before cold provocation with laser Doppler registration was repeated. TNS was performed with a CEFAR stimulator giving 100 Hz square pulses at a frequency of 2 Hz, with a stimulus intensity three times the perception threshold, applied with a cutaneous surface electrode at the 1.dorsal interosseum of the right hand. They were also given a TNS stimulator with detailed instructions, to use for 20 min 3-4 times daily and pro-

Correspondence: M. Skard Heier, Department of Neurology, Ullevål University Hospital, 0407 Oslo 4, Norway.
Received 6 September 1990; and in revised form 12 November 1990.

phylactically before exposure to cold, for a period of 4 weeks.

*Nerve conduction velocities*

Measurements of nerve conduction velocities (NCV) were performed with a DISA Neuromatic 2000, using surface electrodes for stimulation and recording, at skin temperature >30°C. Motor NCV was measured in the right median and anterior peroneal nerves and sensory NCV in the right median and sural nerves.

### Results

*Raynaud's phenomenon*

Laser Doppler in the control group showed a baseline flux with vasomotor oscillations with a frequency of 5–10 $s^{-1}$, reflecting the normally occurring bursts of sympathetic vasomotor impulses. The flux level was about six units. After submersion of the hand in cold water, there was a mean flux reduction of 24%, with a mean restitution time of 1.5 min before baseline flux was regained (Figure 1).

All the eight patients who had received CVB treatment, described typical Raynaud's phenomenon that was first observed 1–3 months after treatment. Six of them still developed Raynaud's phenomenon on exposure to cold, whereas the condition had improved in the other two. No patients had trophical skin changes. None of the patients with Raynaud's phenomenon had rheumatic diseases.

In the CVB treated patients, the mean flux level before cold provocation was similar to the control group, with normal vasomotor oscillations. The mean flux reduction on cold provocation was 61% (range 44–85%) with disappearance of the vasomotor oscillations, and a mean restitution time of >7 min (range 2–>15 min) (Figure 2). In the patients without chemotherapy the findings were similar to the control group.

After 25 min TNS given to six patients, the flux reduction after cold provocation was slightly less in two patients and unchanged in four, whereas the restitution time was unchanged in all. An immediate effect of TNS on peripheral microcirculation could therefore not be demonstrated. After 4 weeks of 25 min TNS 3–4 times daily, none of the patients could report a marked effect on the Raynaud's phenomena, although three patients thought they occurred a little less frequently.

*Polyneuropathy*

Of the eight patients with Raynaud's phenomenon, three had no clinical or neurophysiological signs of polyneuropathy. Five patients had pathological neurophysiological findings, with clinical findings in four. The main symptoms were numbness and paresthesia of the feet, and reduced flexion and extension of the toes. The main clinical findings were abolished achilles tendon reflexes and reduced distal perception of vibration and pain. All the five patients with polyneuropathy had pathological neurophysiological findings in the sural nerve, with reduced sensory nerve action potential amplitude in three, and no measurable potential in two patients. In the other nerves the findings were few and insignificant.

### Discussion

Raynaud's phenomenon is divided into two pathophysiological groups: (1) obstructive Raynaud's phenomenon, with structural changes of the vessel walls and reduced lumen, which is occluded by a normal vasoconstrictor response to cold, and (2) vasospastic Raynaud's phenomenon, with normal caliber of the vessels (Keenan & Porter, 1983; Lafferty *et al.*, 1983).

**Figure 1** Laser Doppler flowmetry of three healthy controls, showing capillary flux in the right index finger before and after cold provocation with submersion of the hand in water holding 15°C for 2 min (arrows).

**Figure 2** Laser Doppler flowmetry of three CVB-treated patients, showing capillary flux in the right index finger before and after submersion of the hand in water holding 15°C for 2 min (arrows), showing an increased and prolonged vasospastic response to cold compared to the normal controls.

In the vasospastic Raynaud's phenomenon the sympathetic vasoconstrictor response to cold is exaggerated and followed by a prolonged period without the normal post sympathetic vasodilatation. An increased activity in sympathetic vasoconstrictor fibres or an increased sensitivity of α- adrenergic receptors in the arteriolar walls have been suggested as mechanisms for the vasoconstrictor component of the Raynaud's phenomenon. The lack of postsympathetic vasodilatation may also be explained by an increased sympathetic tone. However, an as yet poorly understood defect in the histaminergic vasodilating system has also been suggested, possibly caused by a reduction of available tissue histamine. Oral administration of $H_1$- and $H_2$- Histamine blockers to normal subjects has induced Raynaud's phenomenon, and supports this theory (Lafferty *et al.*, 1983). Others have found an increased resistance to the vasodilator effects of prostaglandines in patients with vasospastic Raynaud's phenomenon (Horrobin *et al.*, 1983).

In previous studies Raynaud's phenomenon has been reported in about 30% of patients after CVB treatment (Hansen *et al.*, 1989; Vogelzang *et al.*, 1981). This is in accordance with the results of the questionnaire study from which our patients were recruited.

In a previous study, finger systolic blood pressure was measured in patients with Raynaud's phenomenon after treatment with CVB (Hansen & Olsen, 1989). In that study an enhanced reduction of finger systolic blood pressure was demonstrated as a response to cooling, indicating an increased vasospastic response to cold. However, the duration of this response was not measured, and the method did not allow a study of the vasomotor oscillations normally occuring in the capillaries.

In our study all the patients who had received chemotherapy had normal digital blood flow with normal vasomotor oscillations before cold provocation and an exaggerated and markedly prolonged response to cold compared to the control groups, with disappearance of vasomotor oscillations, emphasising the characteristics of a vasospastic type of Raynaud's phenomenon.

In previous studies (Vogelzang, 1981) there has been no significant difference in the frequency of neuropathy between the patients with and without Raynaud's phenomenon. The mechanisms for development of Raynaud's phenomena are probably different from the pathophysiological mechanisms of the polyneuropathy, as the Raynaud's phenomenon developed about 3–6 months after chemotherapy, when most of the symptoms of autonomic dysfunction and polyneuropathy had disappeared. In our study, three of the eight patients with Raynaud's phenomenon had no evidence of polyneuropathy. The number of patients in the present pilot study was, however, too small for a statistical evaluation.

None of our patients had any convincing effect of TNS, although this treatment has been reported to reduce the vasospastic Raynaud's phenomenon in other studies, possibly by an increased release of the neurotransmitter VIP (vasoactive intestinal polypeptide) (Kaada *et al.*, 1984). This suggests that the Raynaud's phenomenon after cisplatin-based chemotherapy may be caused by other pathophysiological mechanisms.

In conclusion, increased and prolonged vasoconstrictor response to cold, which can be measured by the laser Doppler method, frequently develops after CVB treatment, causing Raynaud's phenomenon of the vasospastic type. Further pathophysiological mechanisms still remain obscure, and a correlation with polyneuropathy as an etiological factor has not been ascertained. As all the previous studies have been retrospective, a prospective study with laser Doppler recordings before, during, and 6–12 months after chemotherapy may give additional information of the development of Raynaud's phenomenon after combination chemotherapy.

### References

AASS, N., KAASA, S., LUND, E., KAALHUS, O., HEIER, M.S. & FOSSÅ, S.D. (1990). Long-term somatic side effects and morbidity in testicular cancer patients. *Br. J. Cancer*, **61**, 151.

CASEY, E.B., JELLIFFE, A.M., LE QUESNE, P.M. & MILLETT, Y. (1973). Vincristine neuropathy. Clinical and neurophysiological manifestations. *Brain*, **96**, 69.

HANSEN, S.W., HELWEG-LARSEN, S. & TROJABORG, W. (1989). Long-term neurotoxicity in patients treated with cisplatin, vinblastine and bleomycin for metastatic germ cell cancer. *J. Clin. Oncol.*, **7**, 1457.

HANSEN, S.W. & OLSEN, N. (1989). Raynaud's phenomenon in patients treated with cisplatin, vinblastine and bleomycin for germ cell cancer: measurement of vasoconstrictor response to cold. *J. Clin. Oncol.*, **7**, 940.

HORROBIN, D.F., JENKINS, K. & MANKU, M.S. (1983). Raynaud's phenomenon, histamine and prostaglandines. *Lancet*, **ii**, 747.

KAADA, B. (1982). Vasodilation induced by transcutaneous nerve stimulation in peripheral ischemia (Raynaud's phenomenon and diabetic polyneuropathy). *Eur. Heart J.*, **3**, 303.

KAADA, B., OLSEN, E. & EIELSEN, O. (1984). In search of mediators of skin vasodilation induced by transcutaneous nerve stimulation: III. Increase in plasma VIP in normal subjects and in Raynaud's disease. *Gen. Pharmacol.*, **15**, 107.

KAPLAN, R.S. & WIERNIK, P.H. (1982). Neurotoxicity of antineoplastic drugs. *Semin. Oncol.*, **9**, 103.

KEENAN, E.J. & PORTER, J.M. (1983). $\alpha_2$ -adrenergic receptors in platelets from patients with Raynaud's syndrome. *Surgery*, **94**, 204.

LAFFERTY, K., ROBERTS, V.C., DE TRAFFORD, J.C. & COTTON, L.T. (1983). On the nature of Raynaud's phenomenon. The role of histamine. *Lancet*, **ii**, 313.

LOW, P.A., NEUMANN, C., DYCK, P.J. & FEALEY, R.D. (1983). Evaluation of skin vasomotor reflexes by using laser doppler flowmetry. *Mayo Clin. Proc.*, **58**, 583.

NILSSON, G.E., TENLAND, T. & ØBERG, P.Å. (1980). Evaluation of laser Doppler flowmeter for measurements of tissue blood flow. *IEEE Trans. Biomed. Eng., BME*, **27**, 597.

ROELOFS, R.I., HRUSHESKY, W., ROGIN, J. & ROSENBERG, L. (1984). Peripheral sensory neuropathy and cisplatin chemotherapy. *Neurology*, **34**, 934.

SALERUD, E.G., TENLAND, T., NILSSON, G.E. & ØBERG, P.Å. (1983). Rythmical variations in human skin blood flow. *Int. J. Microcirc. Clin. Exp.*, **2**, 91.

THOMPSON, S.W., DAVIS, L.E., KORNFELD, M., HILGERS, R.D. & STANDEFER, J.C. (1984). Cisplatin neuropathy. Clinical, electrophysiologic, morphologic and toxicologic studies. *Cancer*, **54**, 1269.

VOGELZANG, N.J., BOSL, G.J., JOHNSON, K. & KENNEDY, B.J. (1981). Raynaud's phenomenon: a common toxicity after combination chemotherapy for testicular cancer. *Ann. Int. Med.*, **95**, 288.

VOGELZANG, N.J., TORKELSEN, J.L. & KENNEDY, B.J. (1985). Hypomagnesemia, renal dysfunction, and Raynaud's phenomenon in patients treated with cisplatin, vinblastine and bleomycin. *Cancer*, **56**, 2765.